# Colonic infusions of short-chain fatty acid mixtures promote energy metabolism in overweight/obese men: a randomized crossover trial

**DOI:** 10.1038/s41598-017-02546-x

**Published:** 2017-05-24

**Authors:** Emanuel E. Canfora, Christina M. van der Beek, Johan W. E. Jocken, Gijs H. Goossens, Jens J. Holst, Steven W. M. Olde Damink, Kaatje Lenaerts, Cornelis H. C. Dejong, Ellen E. Blaak

**Affiliations:** 1 Department of Human Biology, NUTRIM ‘School of Nutrition and Translational Research in Metabolism’, Maastricht University Medical Centre, 6229ER Maastricht, The Netherlands; 2 Top Institute Food and Nutrition, 6700AN Wageningen, The Netherlands; 3 Department of Surgery, NUTRIM ‘School of Nutrition and Translational Research in Metabolism’, Maastricht University Medical Centre, 6229ER Maastricht, The Netherlands; 4 NNF Center for Basic Metabolic Research, Department of Biomedical Sciences, The Panum Institute, University of Copenhagen, 2200 Copenhagen, Denmark; 5 Department of HPB Surgery and Liver Transplantation, Institute of Liver and Digestive Health, University College London, WC1 6HX London, United Kingdom; 6 Department of Surgery, Universitätsklinikum Aachen, 52074 Aachen, Germany

## Abstract

Short-chain fatty acids (SCFA), formed by microbial fermentation, are believed to be involved in the aetiology of obesity and diabetes. This study investigated the effects of colonic administration of physiologically relevant SCFA mixtures on human substrate and energy metabolism. In this randomized, double-blind, crossover study, twelve normoglycaemic men (BMI 25–35 kg/m^2^) underwent four investigational days, during which SCFA mixtures (200 mmol/L) high in either acetate (HA), propionate (HP), butyrate (HB) or placebo (PLA) were rectally administered during fasting and postprandial conditions (oral glucose load). Before and for two hours after colonic infusions, indirect calorimetry was performed and blood samples were collected. All three SCFA mixtures increased fasting fat oxidation (*P* < 0.01), whilst resting energy expenditure increased after HA and HP compared with PLA (*P* < 0.05). In addition, all three SCFA mixtures increased fasting and postprandial plasma peptide YY (PYY) concentrations, and attenuated fasting free glycerol concentrations versus PLA (*P* < 0.05). Colonic infusions of SCFA mixtures, in concentrations and ratios reached after fibre intake, increased fat oxidation, energy expenditure and PYY, and decreased lipolysis in overweight/obese men. Human intervention studies are warranted to investigate whether these effects translate into long-term benefits for body weight control and insulin sensitivity in the obese insulin resistant state.

## Introduction

A growing body of evidence suggests that the gut microbiota has a crucial role in the regulation of energy and substrate metabolism and, as such, in the aetiology of cardiometabolic disease^1^. The gut microbiota ferment indigestible food components, such as dietary fibres, resulting in the production of important metabolites, including short-chain fatty acids (SCFA), which may affect host metabolism^2^.

Over the last decades, a number of studies have proposed that increased fibre content in our daily diet might prevent weight gain and disturbances in glucose and lipid metabolism^2, 3^. However, the underlying mechanisms involved are, so far, not completely understood.

Increasing evidence supports an important role of SCFA, including acetate, propionate and butyrate, in control of body weight and insulin sensitivity^2^. These SCFA might have pronounced effects on host metabolism through the secretion of gut-derived signalling hormones^4–6^ or by entering the systemic circulation^7^, thereby affecting peripheral energy and substrate metabolism. Indeed, several *in vitro* and animal studies have indicated that SCFA are important regulators of energy homeostasis and glucose metabolism^2, 8^. Cell culture studies showed that acetate, propionate and butyrate might alter adipose tissue function, by attenuating intracellular lipolysis^9, 10^, decreasing the production of proinflammatory molecules^10, 11^, as well as by stimulating adipogenesis^12^. Furthermore, it has been shown that oral administration of butyrate affects body weight control via enhanced energy expenditure and fat oxidation in obese mice^13^. In addition, oral administration of acetate, propionate and butyrate to high-fat diet-fed mice all prevented gains in body weight and improved insulin sensitivity without changing energy intake and the amount of physical activity^8, 14, 15^. Based on these rodent data, it is tempting to speculate that colonic administration of SCFA may also have beneficial effects on human substrate and energy metabolism. However, there is also conflicting literature present, i.e. data derived from a rodent study suggested that an increased acetate turnover promote the development of obesity and insulin resistance^16^.

Human data indicating *in vivo* metabolic effects of SCFA are scarce. We have recently demonstrated that acute infusions of the most abundant SCFA acetate in the distal, but not in the proximal, part of the colon enhanced fat oxidation and circulating levels of the satiety-stimulating hormone peptide YY (PYY) in overweight men, indicating an improved metabolic profile^17^. In the present study, we therefore, rectally administered physiologically relevant SCFA mixtures, either high in acetate, propionate or butyrate, and aimed to elucidate the role of gut-derived SCFA on fat oxidation and energy expenditure in overweight/obese normoglycaemic men during fasting and postprandial conditions. Secondary outcomes were effects of SCFA mixtures on carbohydrate oxidation, circulating metabolites (triacylglycerol (TAG), free fatty acids (FFA), free glycerol, glucose, lactate) and hormones (insulin, PYY, glucagon-like peptide 1 (GLP-1), angiopoietin-like protein 4 (ANGPTL4)), plasma SCFA, inflammatory markers (tumour necrosis factor-alpha (TNF-α), interleukin-1-beta (IL-1β), interleukin-6 (IL-6), interleukin-8 (IL-8)) and Visual Analogue Scale (VAS)-scores for hunger and satiety during fasting and postprandial conditions.

## Results

Thirteen overweight/obese normoglycaemic men were included in this trial, of which 12 completed all four clinical investigation days (CID). One participant decided to withdraw from the study before the start of the first CID (Supplementalary Fig. 2). At baseline, the included volunteers had an average age of 36 ± 3 years, a body mass index (BMI) of 30.3 ± 0.8 kg/m^2^ and were normoglycaemic (fasting glucose 5.1 ± 01 mmol/L, HbA1c 5.2 ± 01%, Table 1). No adverse events occurred.

**Table 1:** Participants’ baseline characteristics.

Variables	Mean	SEM
Age (years)	36	3
Height (cm)	180.8	1.4
Weight (kg)	98.8	3.0
BMI (kg/m^2^)	30.3	0.8
Waist circumference (cm)	108	3
Hip circumference (cm)	110	2
Systolic blood pressure (mmHg)	124	2
Diastolic blood pressure (mmHg)	81	1
HbA1c (%)	5.2	0.1
HbA1c (mmol/mol)	33	0.8
Fasting glucose (mmol/L)	5.1	0.1
ALAT (U/L)	35	4
Creatinine (μmol/L)	90	3

*n* = 12; Values are represented as mean ± standard error of mean (SEM); BMI, body mass index; HbA1c, hemoglobin 1Ac; ALAT, alanine-aminotransferase.

### Energy expenditure and substrate oxidation

**Figure 1:**
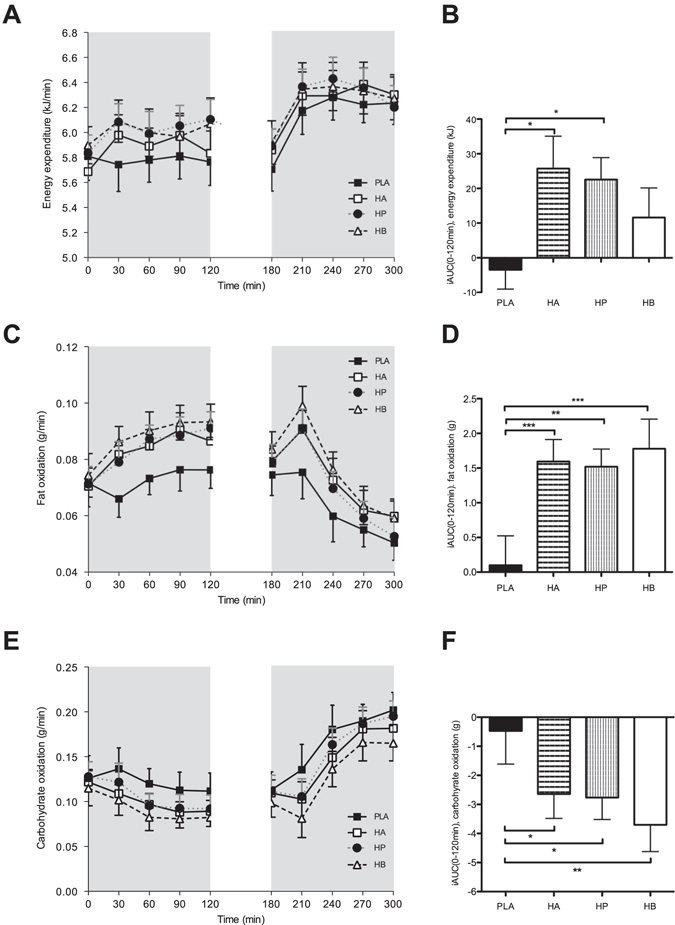
Effect of colonic administration of SCFA mixtures on fasting and postprandial energy expenditure (**A,B**), fat oxidation (**C,D**), and carbohydrate oxidation (**E,F**) (**A**) Resting (t0–t120 min) and postprandial (t180–t300 min) energy expenditure after colonic SCFA infusions. (**B**) iAUC for resting (t0–t120 min) energy expenditure following colonic SCFA infusions. Overall treatment effect for resting energy expenditure *P* = 0.049 (period *P* = 0.933, carry-over *P* = 0.571). (**C**) Fasting (t0–t120 min) and postprandial (t180–t300 min) fat oxidation after colonic SCFA infusions. (**D**) iAUC for fasting (t0–t120 min) fat oxidation following colonic SCFA infusions. Overall treatment effect for fasting fat oxidation *P* < 0.001 (period *P* = 0.325, carry-over *P* = 0.235). (**E**) Fasting (t0–t120 min) and postprandial (t180–t300 min) carbohydrate oxidation after colonic SCFA infusions. (**F**) iAUC for fasting (t0–t120 min) carbohydrate oxidation following colonic SCFA infusions. Overall treatment effect for fasting carbohydrate oxidation *P* = 0.010 (period *P* = 0.315, carry-over *P* = 0.154). Values are means ± SEMs (n = 12). Statistical significance indicated as asterisk (*) when ***P < 0.001, **P < 0.01, *P < 0.05.

Under fasting conditions, energy expenditure increased following a colonic infusion of SCFA mixture high in acetate (HA) and a SCFA mixture high in propionate (HP) as compared to placebo (PLA) (*P* < 0.05, Fig. 1A and B). No significant differences in energy expenditure between interventions were observed during postprandial conditions (Fig. 1A).

All SCFA mixtures (HA, HP and a SCFA mixture high in butyrate (HB)) increased fasting fat oxidation compared with PLA (*P* < 0.01, Fig. 1C and D), which was accompanied by a decreased carbohydrate oxidation (*P* < 0.05, Fig. 1E and F). In accordance, fasting respiratory quotient was decreased with all SCFA mixtures compared to PLA (*P* < 0.05, Supplemental Fig. 3A and B). During the postprandial phase, fat and carbohydrate oxidation rates were not significantly different between interventions (Fig. 1C and E).

### Plasma analysis

#### Plasma short-chain fatty acid concentrations

**Figure 2:**
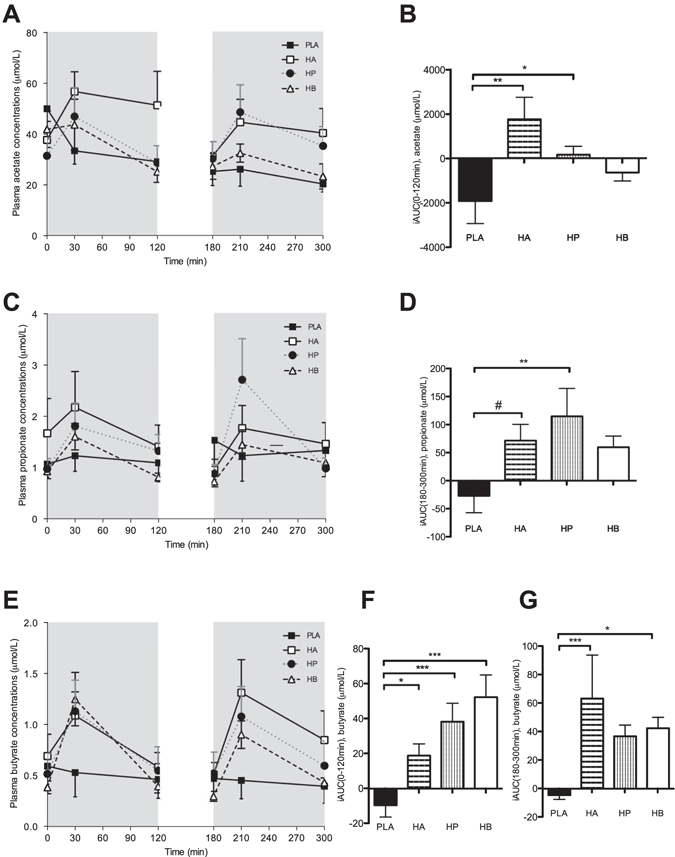
Effect of colonic administration of SCFA mixtures on fasting and postprandial plasma acetate (**A,B**), propionate (**C,D**), and butyrate (**E–G**) concentrations (**A**) Fasting (t0–t120 min) and postprandial (t180–t300 min) plasma acetate concentrations after colonic SCFA infusions. (**B**) iAUC for fasting (t0–t120 min) plasma acetate following colonic SCFA infusions. Overall treatment effect for fasting plasma acetate *P* = 0.051 (period *P* = 0.338, carry-over *P* = 0.898). (**C**) Fasting (t0–t120 min) and postprandial (t180–t300 min) plasma propionate concentrations after colonic SCFA infusions. (**D**) iAUC for postprandial (t180–t300 min) plasma propionate following colonic SCFA infusions. Overall treatment effect for postprandial plasma propionate *P* = 0.068 (period *P* = 0.781, carry-over *P* = 0.896). (**E**) Fasting (t0–t120 min) and postprandial (t180–t300 min) plasma butyrate concentrations after colonic SCFA infusions. (**F**) iAUC for fasting (t0–t120 min) plasma butyrate following colonic SCFA infusions. Overall treatment effect for fasting plasma butyrate *P* < 0.001 (period *P* = 0.997, carry-over *P* = 0.879) (**G**) iAUC for postprandial (t180–t300 min) plasma butyrate following colonic SCFA infusions. Overall treatment effect for postprandial plasma butyrate *P* = 0.032 (period *P* = 0.754, carry-over *P* = 0.971). Values are means ± SEMs (n = 12). Statistical significance indicated as asterisk (*) when ***P < 0.001, **P < 0.01, *P < 0.05 and as hashtag when ^#^ *P* < 0.10.

HA and HP mixtures increased fasting plasma acetate concentrations compared with PLA (*P* < 0.05, Fig. 2A and B). During the postprandial phase, acetate concentrations were not significantly different between treatments (Fig. 2A).

No significant treatment effect was observed on circulating propionate concentrations in the fasting period (Fig. 2C). However, in the postprandial period plasma propionate concentrations were increased with HP treatment as compared to PLA (*P* = 0.008, Fig. 2C and D).

Under fasting conditions, plasma butyrate concentrations increased following administration of all SCFA mixtures, when compared to PLA (*P* < 0.05, Fig. 2E and F). Postprandial circulating butyrate concentrations increased after HA and HB treatment, when compared to PLA (*P* < 0.05, Fig. 2E and G).

#### Associations between plasma SCFA concentrations and energy and fat oxidation

The increments of fasting plasma acetate concentrations were positively correlated with the increments of resting energy expenditure (r = 0.349, *P* = 0.0149, supplementary Fig. 4A) and fasting fat oxidation (r = 0.328, *P* = 0.0228, supplementary Fig. 4B). Changes in plasma propionate and butyrate concentrations did not correlate with changes in fat oxidation or resting energy expenditure, neither in the fasting nor in the postprandial period.

#### Plasma metabolites and insulin concentrations

**Figure 3:**
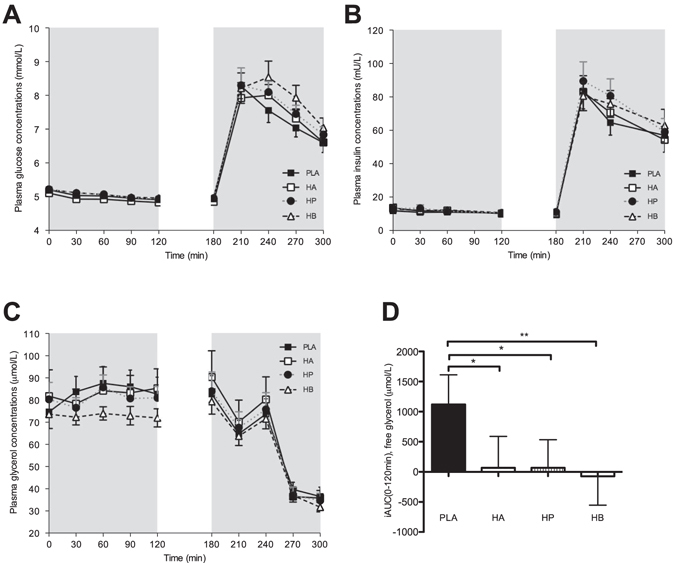
Effect of colonic administration of SCFA mixtures on fasting and postprandial plasma glucose (**A**), insulin (**B**) and free glycerol (**C,D**) concentrations (**A**) Fasting (t0–t120 min) and postprandial (t180–t300 min) plasma glucose concentrations after colonic SCFA infusions. (**B**) Fasting (t0–t120 min) and postprandial (t180–t300 min) plasma insulin concentrations after colonic SCFA infusions. (**C**) Fasting (t0–t120 min) and postprandial (t180–t300 min) plasma free glycerol concentrations after colonic SCFA infusions. (**D**) iAUC for fasting (t0–t120 min) plasma free glycerol following colonic SCFA infusion. Overall treatment effect for free glycerol *P* = 0.030 (period *P* = 0.483, carry-over *P* = 0.832). Values are means ± SEMs (n = 12). Statistical significance indicated as asterisk (*) when **P < 0.01, *P < 0.05.

Fasting and postprandial plasma glucose concentrations did not differ between treatments. Although, postprandial plasma glucose and insulin concentrations were elevated following administration of all SCFA mixtures and PLA, no significant treatment effect was observed (Fig. 3A and B). Fasting plasma lactate concentrations did not differ between treatments (supplementary Fig. 5A). However, postprandial lactate concentrations increased after HP mixture infusions as compared to HB and PLA (*P* < 0.05, supplementary Fig. 5A and B).

There were no significant differences in circulating plasma TAG concentrations between the interventions, neither in the fasting nor in the postprandial period (supplementary Fig. 5C). The administration of all three SCFA combinations decreased circulating fasting free glycerol concentrations compared with PLA (*P* < 0.05, Fig. 3C and D). Postprandial free glycerol concentrations decreased in all intervention groups, with no significant differences between interventions (Fig. 3C). No significant treatment effect was observed on circulating FFA concentrations in the fasting and postprandial period (supplementary Fig. 5D).

#### Plasma PYY, GLP-1 and ANGPTL4 concentrations

**Figure 4:**
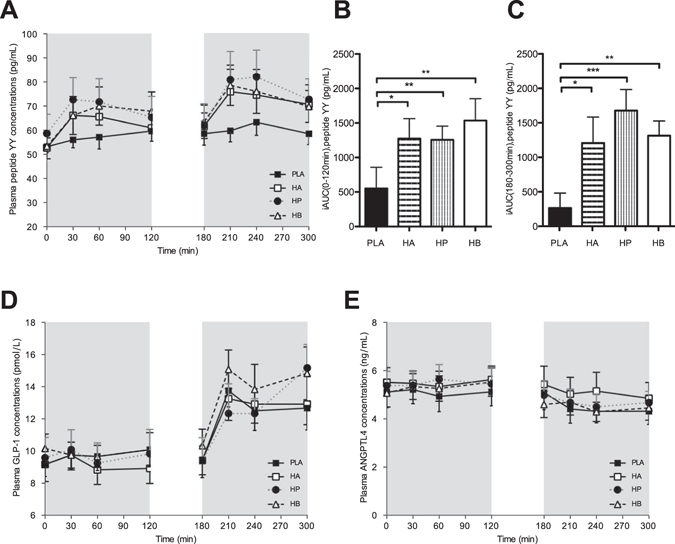
Effect of colonic administration of SCFA mixtures on fasting and postprandial plasma PYY (**A–C**), GLP-1 (D) and ANGPTL4 (F) (**A**) Fasting (t0–t120 min) and postprandial (t180–t300 min) plasma PYY concentrations after colonic SCFA infusions. (**B**) iAUC for fasting (t0–t120 min) plasma PYY following colonic SCFA infusion. Overall treatment effect fasting PYY *P* = 0.011 (period *P* = 0.557, carry-over *P* = 0.553). (**C**) iAUC for postprandial (t120–t300 min) plasma PYY following colonic SCFA infusion. Overall treatment effect postprandial PYY *P* < 0.001 (period *P* = 0.595, carry-over *P* = 0.184). (**D**) Fasting (t0–t120 min) and postprandial (t180–t300 min) plasma GLP-1 concentrations after colonic SCFA infusions. (**E**) Fasting (t0–t120 min) and postprandial (t180–t300 min) plasma ANGTPL4 concentrations after colonic SCFA infusions. Values are means ± SEMs (n = 12). Statistical significance indicated as asterisk (*) when ***P < 0.001 and **P < 0.01 and *P < 0.05.

PYY was increased with all SCFA combinations (HA, HP and HB) both during fasting and postprandial conditions as compared to placebo (*P* < 0.05, Fig. 4A,B and C).

Fasting GLP-1 levels showed no significant differences after infusion of SCFA mixtures. Postprandial GLP-1 concentrations increased following colonic administration of SCFA mixtures and PLA, without significant differences between groups (Fig. 4C).

ANGPTL4 concentrations did not show statistically significant differences between treatments neither in the fasting nor in the postprandial period (Fig. 4D).

#### Plasma concentrations of inflammatory markers

**Figure 5:**
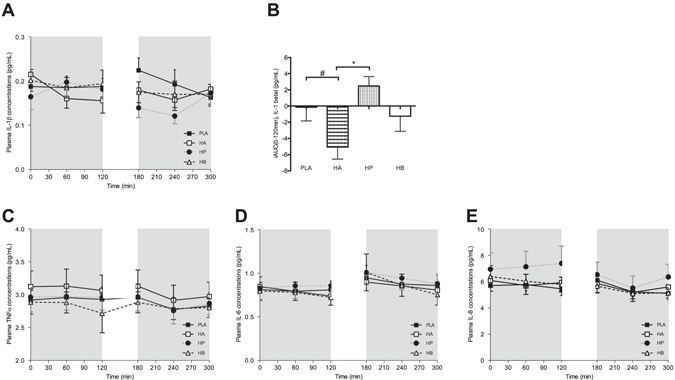
Effect of colonic administration of SCFA mixtures on fasting and postprandial plasma inflammatory cytokines (**A**) Fasting (t0–t120 min) and postprandial (t180–t300 min) plasma IL-1β concentrations after colonic SCFA infusions. (**B**) iAUC for fasting (t0–t120 min) plasma IL-1β following colonic SCFA infusion. Values are means ± SEMs (n = 10, for two volunteers IL-1β concentrations were not detectable). Overall treatment effect for fasting IL-1β *P = *0.068 (period *P* = 0.626, carry-over *P* = 0.841). (**C**) Fasting (t0–t120 min) and postprandial (t180–t300 min) plasma TNF-α after colonic SCFA infusions. Values are means ± SEMs (n = 12).) (**D**) Fasting (t0–t120 min) and postprandial (t180–t300 min) plasma IL-6 after colonic SCFA infusions. Values are means ± SEMs (n = 11, for one participant IL-6 concentrations were not detectable). (**E**) Fasting (t0–t120 min) and postprandial (t180–t300 min) plasma IL-8 after colonic SCFA infusions. Values are means ± SEMs (n = 12). Statistical significance indicated as asterisk (*) when *P < 0.05 and as hashtag when ^#^ *P* < 0.10.

Fasting circulating levels of the proinflammatory cytokine IL-1β decreased with HA, when compared with HP (*P* < 0.05, Fig. 5A and B). Postprandial IL-1β levels did not differ between treatments (Figure A).

Other proinflammatory cytokines including TNF-α, IL-6 and IL-8 were not affected by SCFA infusion neither in the fasting nor in the postprandial period (Fig. 5C,D and E).

Hunger and satiety scores: Overall, there were no significant differences in VAS scores between interventions, neither in the fasting nor in the postprandial period (Supplement Fig. 6).

## Discussion

In the present randomized, double-blind, placebo-controlled crossover study, the effects of administration of three different SCFA mixtures in the distal part of the colon on fat oxidation, energy expenditure and metabolic parameters were assessed in twelve overweight or obese, normoglycaemic men. We demonstrated that infusions of colonic SCFA mixtures increase fasting fat oxidation and resting energy expenditure, which was correlated with increments in circulating acetate concentrations. In addition, these SCFA mixtures increased fasting and postprandial PYY concentrations, and attenuated whole-body lipolysis, as indicated by decreased free glycerol concentrations.

The ratios, absolute amounts and concentrations of SCFA used in this human *in vivo* study represent colonic physiological mixtures, which putatively can be achieved in the colon by the intake of a diet rich in dietary fibres^18–20^. The human gut microbiota ferments orally ingested complex carbohydrates into a combination of acetate, propionate and butyrate in the lumen of the distal gut^21^. The exact ratios and amounts produced are dependent on many factors, including the type of fermented carbohydrate, the specific microbial species, diversity and absolute abundance of host’s gut microbiota, and colonic transit time^18, 19, 21^. Theoretically, the total amount of SCFA produced over the whole colon can be up to 800 mmol per day^2, 22^. Data from six suddenly deceased individuals showed SCFA concentrations up to ~190 mmol/kg of luminal content in the distal part of the colon^23^. In the present study, SCFA concentrations of 200 mmol/L (40 mmol in a 200 mL solution) were administered rectally. In addition, the use of colonic SCFA mixtures containing all three SCFA is a better representation of human physiological conditions than administering single SCFA as has been done in previous studies^17, 24, 25^.

The effects of the SCFA mixtures on fat oxidation and energy expenditure found in the present study suggest an important role for gut-derived SCFA in whole-body energy metabolism. This might be one of the explanations for the beneficial effects of dietary fibre(s) on body weight control and glucose homeostasis on the long term^2, 3^. It is important to mention that oxidation of the total amount of infused sodium acetate only contributes to a minor extent to the observed increase in fat oxidation, as we previously calculated and discussed extensively elsewhere^17^. Next, in human *in vivo* studies with rectal and caecal administration of isotopically-labelled butyrate showed that only ~25% and ~33% of butyrate is oxidized within a 4 hours or a 12 hours timeframe, respectively^26, 27^. In addition, a study showed that rectally infused butyrate (5 mmol) did not change whole-body respiratory quotient and resting energy expenditure^26^. Furthermore, propionate and butyrate oxidation yield a respiratory quotient of 0.86 and 0.8, respectively^28^. This demonstrates that also propionate utilization would contribute only modestly to the effects on whole-body fat oxidation in our study. Together, these findings indicate that oxidation of infused SCFA mixtures are unlikely to explain the observed increase in fasting whole-body fat oxidation and energy expenditure in the present study.

Based on mainly rodent data, several putative other mechanisms are expected to explain these SCFA-induced increases in fat oxidation and energy expenditure. Den Besten *et al*.^14^ showed that orally administered acetate, propionate and butyrate prevented high-fat diet induced weight gain and improved glucose metabolism in mice, without alterations in food intake and physical activity. Moreover, they observed a peroxisome proliferator-activated receptor-γ (PPARγ)-dependent increase in the expression of mitochondrial uncoupling protein 2 and elevations of the AMP/ATP ratio. This led to a switch from lipid synthesis to fat oxidation via an adenosine monophosphate-activated protein kinase (AMPK)-dependent mechanism in liver and adipose tissue ^14^. In addition, Gao and colleagues ^13^ found that oral butyrate administration reduced adiposity and increased insulin sensitivity in obese mice, without a change in food intake, fat absorption and locomotor activity. These findings were explained by increased energy expenditure and fat oxidation via activation of AMPK and (PPARγ)-coactivator (PGC)-1α in the liver and the skeletal muscle ^13^. Along the same line, Yashamita *et al*. ^29^ found that intragastric injections of acetate reduced body weight and improved glucose homeostasis in obese rats via an increase in fat oxidation mediated by an increased AMPK activity in the skeletal muscle ^29^. However, whether an AMPK-mediated enhanced oxidative metabolism and the mobilization of intracellular lipids in peripheral tissue underlie the present findings in human remains to be established.

Of note, we found that the HA and HP mixtures increased circulating acetate concentrations and that all SCFA mixtures increased circulating butyrate concentrations during fasting conditions. In addition, the HP mixtures increased postprandial circulating propionate concentrations. Nevertheless, only the increments in fasting acetate concentrations correlated positively with the increments in fasting fat oxidation and resting energy expenditure, suggesting that circulating acetate is the major driver of these metabolic effects in humans. Consistent with this observation, we previously observed a comparable increase in fat oxidation (~25%) in overweight to obese men after distal colonic administration of sodium acetate alone, when compared to a saline infusion ^17^. Based on available animal data ^8, 14, 29, 30^ and the increased systemic acetate concentrations found in the present study, it might be speculated that the circulating acetate leads to an increased oxidative capacity and a substrate switch to fat utilization in the liver and peripheral organs such as skeletal muscle. However, so far no human data of SCFA effects on human skeletal muscle and liver oxidative metabolism are available, which therefore have a high degree of interest for future studies.

In addition, all SCFA mixtures increased plasma PYY concentrations. Besides its well-described ability to act as a satiety–stimulating hormone ^31–33^, studies in mice and preliminary human data suggest that an increase in circulating PYY shifts substrate utilization towards increased fat oxidation ^34, 35^. Therefore, the increased systemic PYY availability found in this study might have also contributed to the increased fat oxidation. However, the underlying mechanisms are not known yet.

Moreover, the present results indicate that the SCFA mixtures slightly decreased fasting plasma glycerol concentrations, indicating a decrease in lipolysis. This is consistent with previous reports showing that single SCFA, in particular acetate, blunt whole-body lipolysis in humans ^24, 36, 37^. Ge *et al*. ^9^ showed that treatment of rodent-derived 3T3-L1 adipocytes with acetate and propionate reduced the intracellular lipolytic activity as assessed by a decreased release of glycerol in the culture medium ^9^, thereby identifying these SCFA as regulators of adipose tissue metabolism. Aberdein *et al*. ^38^ found that an acetate-induced decreased hormone-sensitive lipase activity might underlie this antilipolytic effect, as they showed in mature 3T3-L1 adipocytes. Data derived from rodents and overweight humans indicate that a partially inhibition of intracellular lipolysis in adipocytes prevent ectopic fat accumulation and insulin resistance in tissues such as the liver and skeletal muscle without affecting adipose tissue mass in the longer term ^39^.

In addition, we found a slight increase in postprandial plasma lactate concentrations after HP. This might be partly explained by a propionate induced inhibition of the pyruvate dehydrogenase complex activity as found in rodent-derived liver cells ^40^. Pyruvate dehydrogenase regulates the decarboxylation of pyruvate and thereby contributes to the conversion from pyruvate to acetyl-CoA, thereby linking the glycolysis pathway to the citric acid cycle under aerobic conditions. A decreased activity of the pyruvate dehydrogenase complex results in increased pyruvate concentrations, which consequences an anaerobic cascade resulting in increased lactate production. However, whether this also occurs in human tissue has to be investigated.

The increase in fat oxidation, together with an increase in the satiety-stimulating gut hormone, PYY, found in the present study, indicate that elevating SCFA, in particular acetate, in the colon and in the circulation, might have important clinical implications on food intake regulation and long-term control of body weight. However, it remains to be determined whether a SCFA-induced increase in PYY concentrations translate into long-term effects on appetite regulation, in particular, since a study using a SCFA-fibre ester was unable to translate acute effects on postprandial PYY levels to the long term in overweight humans ^41^.

Furthermore, SCFA (acetate)-induced fat oxidation and/or increased oxidative capacity in skeletal muscle might improve metabolic flexibility, defined as the capacity to utilize and switch between the major fuels, lipids and glucose ^42^. Together with an acetate-induced partial inhibition of intracellular lipolysis in adipocytes this might result in reduced fat accumulation and improved insulin action in peripheral tissues such as skeletal muscle, liver and pancreas, thereby preventing insulin resistance.

Most luminal SCFA production occurs in the cecum and proximal part of the colon, where substrate availability is highest ^43^. Of interest, we found in our previous study that acetate administration in the distal rather than the proximal part of the colon evoked metabolic effects ^17^. We hypothesized that an increased systemic acetate availability after distal colonic installation, explained these differential effects. SCFA delivered in the distal colon partly bypass the liver via the rectal venous plexus, which drains into the inferior vena cava, thereby directly reaching the systemic circulation and increasing the SCFA availability in metabolically active peripheral organs such as skeletal muscle and adipose tissue. Specific slow-fermentable acetogenic nutrients like fibre-SCFA ester, which was previously used to deliver propionate to the colon ^41, 44^, and/or the delivery of homoacetogenic bacteria to the distal colon might lead to an increased production of acetate in the descending and sigmoid colon, and hence be an interesting approach for the control of body weight and glucose homeostasis.

In conclusion, the present study demonstrated that acute rectal administration of putatively physiological concentrations of SCFA modulates whole-body substrate and energy metabolism, with an increase in fasting fat oxidation and resting energy expenditure, which was associated with an increase in circulating acetate concentrations. In addition, these SCFA mixtures increased fasting and postprandial concentrations of the satiety-stimulating hormone PYY, and attenuated whole-body lipolysis. Human intervention studies are warranted to investigate whether these effects translate into benefits for body weight control and insulin sensitivity in the long term and to provide evidence that increasing colonic SCFA levels might be a strategy to prevent and/or reverse the obese insulin resistant state.

## Material and Methods

### Study participants

Thirteen overweight and obese (BMI 25–35 kg/m^2^), 20–50 years old normoglycaemic Caucasian men were recruited between August 2013 and January 2014 from the vicinity of Maastricht, the Netherlands. Exclusion criteria were the presence of diabetes mellitus (defined as fasting plasma glucose ≥ 7.0 mmol/L), gastroenterological diseases or prior abdominal surgery, cardiovascular diseases, cancer, liver or kidney malfunction, a life expectancy shorter than 5 years, use of a hypocaloric diet, use of laxatives, or use of antibiotics, pre- or probiotics in the 3 months prior to the start of the study or during the study period. The study was approved by the Medical Ethical Committee of Maastricht University Medical Centre (MUMC + ) and conducted in accordance with the Declaration of Helsinki (revised version, October 2008, Seoul, South Korea). Written informed consent was obtained from all participants. All authors had access to the study data and reviewed and approved the final manuscript.

### Study design and randomization

This study was performed using a double-blind, placebo-controlled, randomized, crossover design. Participants were studied during four CID, each with at least a five-day washout period in between. During each CID the participants received either one of the three colonic SCFA mixtures infusions or a placebo infusion via enemas after an overnight fast of at least 12 hours. Three hours after the first rectal infusion, a second enema was administered after an oral glucose load of 75 gram, which resembled the postprandial state. The order of solution administration was blinded for both the investigator and participants. An independent researcher performed permuted block randomization and assigned participants to interventions.

### Investigational products

In this study all participants received enemas containing HA, HP or HB or they received PLA in a randomized order. The HA solution contained 24 mmol sodium acetate (60%), 8 mmol sodium propionate (20%), 8 mmol sodium butyrate (20%) dissolved in 200 mL sterile water. The HP solution contained 18 mmol sodium acetate (45%), 14 mmol sodium propionate (35%), 8 mmol sodium butyrate (20%) dissolved in 200 mL sterile water. The HB solution contained 18 mmol sodium acetate (45%), 8 mmol sodium propionate (20%), 14 mmol sodium butyrate (35%) dissolved in 200 mL sterile water. As a PLA, 40 mmol sodium chloride dissolved in 200 mL sterile water was administered. All solutions were isosmotic and equivalent in sodium content.

The SCFA mixtures and sodium chloride were provided pre-weighed in powder form by Basic Pharma Technologies B.V (Geleen, The Netherlands) and were freshly dissolved in the appropriate amount of sterile water (delivered with the SCFA) on the morning of the test days by an independent person. All solutions were produced in accordance with standards of European Good Medical Practice (GMP) requirements.

### Clinical investigation days

Two days prior to the CID, participants were asked to refrain from intense physical activity and alcohol consumption. In the evening before each CID, the volunteers consumed a standardized low fibre meal (57 energy% carbohydrate, 24 energy% proteins and 19 energy% fat). Participants came to the laboratory by car or bus in the morning after an overnight fast (12 h). Each of the four CID consisted of two periods of each 2 h: a fasting period (t0–t120 min) and a subsequent postprandial period (t180–t300 min) (Supplemental Fig. 1). Between the fasting and postprandial periods, no measurements took place for 30 minutes. Prior to the start of each CID (after an overnight fast), a cannula was placed in an antecubital vein of the forearm to enable venous blood sampling. Each period was preceded by venous blood sampling (at t0 for the fasting and at t180 for the postprandial period), completion of a VAS recording for hunger and satiety and measurements of energy expenditure and substrate oxidation for 30 minutes (from t-30–t0 before the fasting period and from t150–t180 before the postprandial period), using an open circuit ventilated hood system (Omnical, MUMC + , The Netherlands^45^).

After baseline measurement, participants inserted a lubricated nozzle of an enema (Blockland BV, Amsterdam, the Netherlands) into the rectum and one of the SCFA mixtures or placebo was manually administered by the research coordinator within 5 min at a rate of 40 mL per minute during fasting conditions (at t = 0), and immediately after the glucose load (at t = 180) in the postprandial period. During the rectal infusions and for exact ten subsequent minutes, the participants remained on the left side-lying position with the knees drawn to the abdomen, to ensure that the infused solution reached the whole distal colon up to the splenic flexure ^46^. Subsequently, in both the fasting and postprandial period, energy expenditure and substrate oxidation were measured for the complete 2 h period. In addition, venous blood was sampled at 30, 60, 90 and 120 minutes after colonic SCFA infusions in both periods. Also, VAS-scores were completed 30, 60, 90 and 120 minutes after colonic administration. Primary outcome was the effect of colonic SCFA mixtures on fat oxidation and energy expenditure during fasting and postprandial conditions. Secondary outcomes were carbohydrate oxidation, circulating metabolites (TAG, FFA, free glycerol, glucose, lactate) and hormones (insulin, active PYY, total GLP-1, ANGPTL4), plasma SCFA, inflammatory markers (TNF-α, IL-1β, IL-6, IL-8) and VAS-scores.

### Blood collection, storage and biochemical analyses

Blood was collected into pre-chilled tubes for insulin, glucose, lactate, FFA, TAG, free glycerol, SCFA, ANGPTL4, TNF-α, IL-1β, IL-6, and IL-8 analysis. For GLP-1 analysis, blood was collected in a 2 mL EDTA tube containing 20 μL of dipeptidyl peptidase-IV inhibitor (Millipore, Darmstadt, Germany). For PYY analysis, blood was collected in a 2 mL aprotinin tube containing 20 μL of dipeptidyl peptidase-IV inhibitor. The samples were centrifuged at 3,500 g, 4 °C for 10 minutes, plasma was aliquoted and directly snap-frozen in liquid nitrogen and stored at −80 °C until analysis.

Plasma FFA, TAG, and glucose were measured with enzymatic assays on an automated spectrophotometer (ABX Pentra 400 autoanalyzer, Horiba ABX, Montpellier, France). Plasma free glycerol and lactate were measured after precipitation with an enzymatic assay automated on a Cobas Fara spectrophotometric autoanalyzer (Roche Diagnostics, Basel, Switzerland). Circulating insulin and PYY concentrations were determined with commercially available radioimmunoassay (RIA) kits (Human Insulin specific RIA, Human PYY (3–36) RIA, Millipore Corporation, MA, USA). IL-1β, IL-6, IL-8 and TNF-α were determined with an enzyme-linked immunosorbent assay (ELISA) kit (Human ProInflammatory II 4-Plex Ultra-Sensitive Kit, Meso Scale Diagnositics, MD, USA). Plasma ANGPTL4 was measured by ELISA as described by Kersten *et al*.^47^. Plasma samples were assayed for total GLP−1 immunoreactivity using an antiserum, which reacts equally with intact GLP-1 and the primary (N-terminally truncated) metabolite, as previously described ^48^.

Deproteinization and subsequent preparation of plasma samples for analysis of SCFA was performed as reported before ^49^. Analysis was performed using a liquid chromatography system combined with mass spectrometry (LC-MS). The detection limits for acetate, propionate and butyrate of this method were 0.1, 0.05 and 0.05 μmol/L, respectively.

### Calculations

The equations of Weir ^50^ and Frayn ^51^ were used to calculate total rate of fat and carbohydrate oxidation and energy expenditure, assuming that protein oxidation represents 15% of total energy expenditure.

### Statistical analysis

A power calculation was based on our recent crossover study with colonic infusions of acetate, using a comparable CID design and a comparable study population ^17^. We calculated, using GPower (Version 3.1 for Mac, Parkville, Victoria, Australia), that 12 participants are sufficient to detect a 20% difference in our primary outcome parameter fat oxidation, with a standard deviation of 5% and a 80% power at an alpha level of *P* = 0.05 and a two-tailed distribution. Considering a potential dropout rate of 5–10% during the protocol, the final number of participants that were recruited was *n* = 13.

Values are expressed as mean ± SEM. Responses after rectal SCFA mixtures and PLA administration during fasting (t0–t120) and postprandial (t180–t300) conditions are expressed as incremental area under the curve (iAUC), which were calculated by the trapezoid method. Histogram and Kolmogorov-Smirnov test were used to check for normality. Differences in fasting and postprandial iAUC between intervention groups were analysed using a linear mixed model for repeated measures. Intervention and period were set as fixed factors and participants were set as random factor. Although no carry-over effects were expected due to a 5-day washout period, we tested for carry-over effects by adding sequence (order of treatments over period) to the model. No correction for multiple testing was made due to the explorative nature of this study. iAUC not shown as values or figures did not present statistically significant differences. Association between plasma SCFA increments and increments of fat oxidation and energy expenditure were tested by linear regression. Statistics were done using SPSS 22.0 for MAC (Chicago, IL, USA). A *P* < 0.10 (two-sided *P*-value) was considered as trend and a *P* < 0.05 (two-sided *P*-value) was considered statistically significant.

## Electronic supplementary material

Supplementary file
