# Supplementary material for: Colonic infusions of short-chain fatty acid mixtures promote energy metabolism in overweight/obese men: a randomized crossover trial
Source: Sci Rep. 2017 May 24;7:2360. doi: 10.1038/s41598-017-02546-x (PMC5443817; doi:10.1038/s41598-017-02546-x)
Supplement: Supplementary file 1 — Supplementary file [file 41598_2017_2546_MOESM1_ESM.docx]

**SUPPLEMENTARY DATA**

**Colonic infusions of short-chain fatty acid mixtures promote energy metabolism in overweight/obese men: a randomized crossover trial**

Emanuel E. Canfora^1,2#^, Christina M. van der Beek^2,3^, Johan W.E. Jocken^1,2^, Gijs H. Goossens^1,2^, Jens J. Holst^4^, Steven W. M. Olde Damink^3,5^, Kaatje Lenaerts^2,3^, Cornelis H.C. Dejong^2,3,6^, Ellen E. Blaak^1,2^

^1^ Department of Human Biology, NUTRIM ‘School of Nutrition and Translational Research in Metabolism’, Maastricht University Medical Centre+, 6229ER Maastricht, The Netherlands

^2^ Top Institute Food and Nutrition, 6700AN Wageningen, the Netherlands

^3^Department of Surgery, NUTRIM ‘School of Nutrition and Translational Research in Metabolism’, Maastricht University Medical Centre+, 6229ER Maastricht, The Netherlands

^4^ NNF Center for Basic Metabolic Research, Department of Biomedical Sciences, The Panum Institute, University of Copenhagen, 2200 Copenhagen, Denmark

^5^Department of HPB Surgery and Liver Transplantation, Institute of Liver and Digestive Health, University College London, WC1 6HX London, United Kingdom

^6^Department of Surgery, Universitätsklinikum Aachen, 52074 Aachen, Germany

**
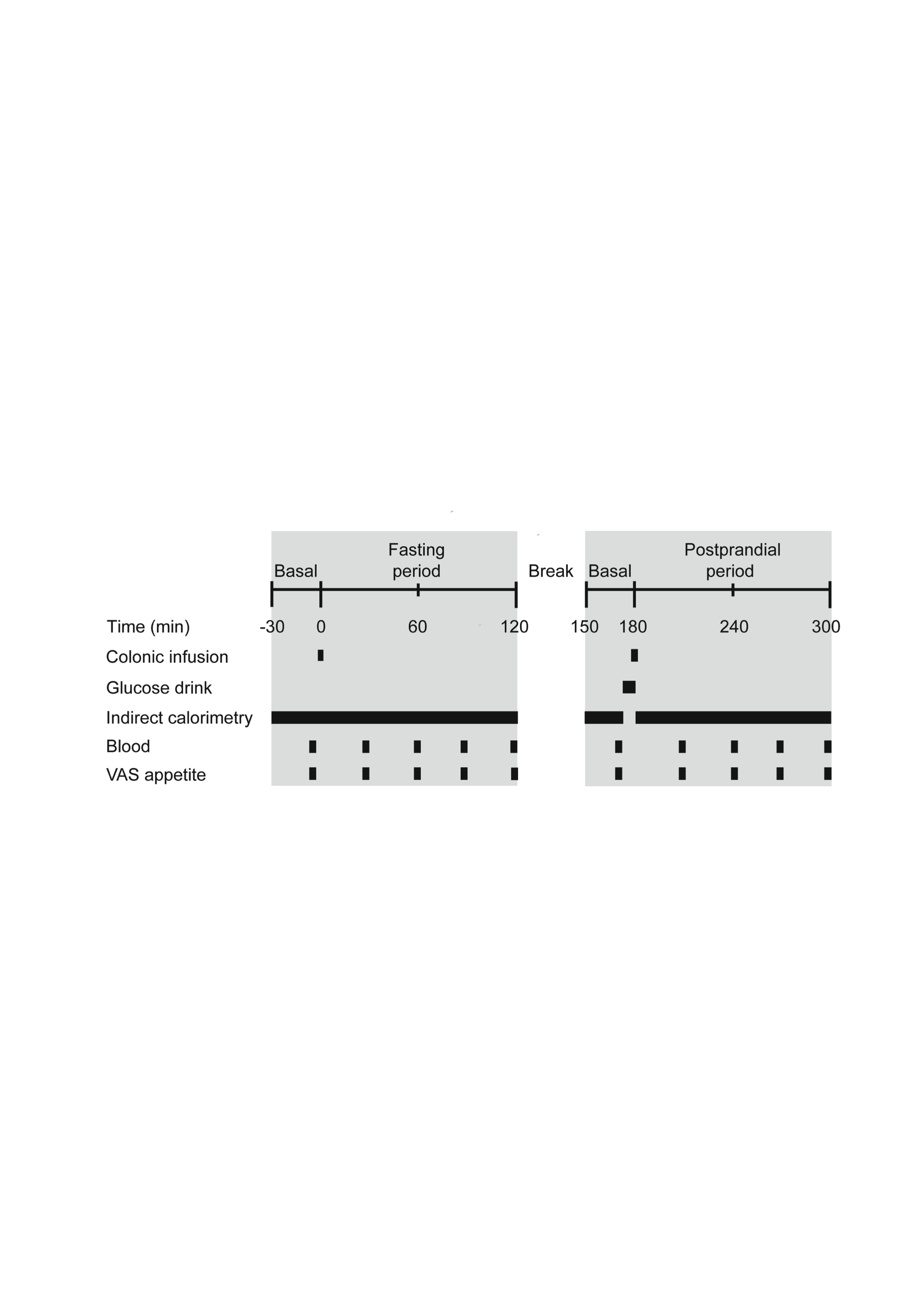
**

**Figure S1. Clinical investigation day protocol**

**Figure S2. Flow diagram of human subjects included in this study.**

**Figure S3**: **A:** **Effect of colonic administration of SCFA mixtures on fasting and postprandial respiratory quotient** Fasting (t0 – t120 min) and postprandial (t180 – t300 min) respiratory quotient after colonic SCFA infusions. **B:** AUC for fasting (t0 – t120 min) respiratory quotient following colonic SCFA infusions. Overall treatment effect for fasting respiratory quotient *P*=.046 (period *P*=.480, carry-over *P*=.592).

Values are means ± SEMs (n = 12). Statistical significance indicated as asterisk (*) when *** *P*<.001, ***P*<.01 and **P*<.05.

**Figure S4:** Relationship between increments of fasting acetate concentrations and increments of fasting fat oxidation and resting energy expenditure. **A:** Acetate vs. fat oxidation r=0.328 (*P*=.0228). **B:** Acetate vs. energy expenditure r=0.349 (*P*=.0149).

**Figure S5: Effect of colonic administration of SCFA mixtures on fasting and postprandial plasma lactate (A, B), TAG (C) and FFA (D) concentrations**

**A:** Fasting (t0 – t120 min) and postprandial (t180 – t300 min) plasma lactate concentrations after colonic SCFA infusions. **D:** iAUC for postprandial (t180 – t300 min) plasma lactate following colonic SCFA infusions. Overall treatment effect for postprandial plasma lactate *P=*.022 (period *P*=.980, carry-over *P*=.994).

**C:** Fasting (t0 – t120 min) and postprandial (t180 – t300 min) plasma TAG concentrations after colonic SCFA infusions.

**D:** Fasting (t0 – t120 min) and postprandial (t180 – t300 min) plasma FFA concentrations after colonic SCFA infusions.

Values are means ± SEMs (n = 12). Statistical significance indicated as asterisk (*) when ****P*<.001, **P*<.05 and as hashtag when #*P*<.10.

**

**Figure S6:** Fasting (t0 – t120 min) and postprandial (t180 – t300 min) VAS for feeling of hunger (as representative for VAS scores hunger and appetite) after colonic SCFA infusions (rating 0 = no feeling of hunger -10 = extreme feeling of hunger). Values are means ± SEMs (n = 12).
